# Amisulpride Augmentation in Schizophrenia Patients with Poor Response to Olanzapine: A 4-week, Randomized, Rater-Blind, Controlled, Pilot Study

**DOI:** 10.1192/j.eurpsy.2023.2322

**Published:** 2023-07-19

**Authors:** W.-M. Bahk, Y. S. Woo, S.-Y. Park, B.-H. Yoon, S.-M. Wang, M.-D. Kim

**Affiliations:** 1 Psychiatry, The Catholic University of Korea, Seoul; 2 Psychiatry, Keyo Hospital, Uiwang; 3 Psychiatry, Naju National Hospital, Naju; 4 Psychiatry, Jeju National University, Jeju, Korea, Republic Of

## Abstract

**Introduction:**

Olanzapine (OLA) is a common first-prescribed antipsychotic and has shown favorable efficacy in acutely exacerbated patients with schizophrenia. The mixed receptor activity of OLA and its greater affinity for serotonin 5-HT2A rather than dopamine D2 receptors are similar to those of clozapine. Pharmacokinetically, OLA is metabolized mainly by hepatic cytochrome enzyme P450 1A2 (CYP1A2). Because risks of antipsychotic polypharmacy include increased drug-drug interactions, pharmacokinetic considerations are important for selection of antipsychotics to be combined. Due to its pharmacological characteristics, amisulpride (AMI), another atypical antipsychotic with proven efficacy, is a promising adjuvant agent of special interest. AMI is unlikely to interact with other drugs due to the low plasma protein binding and metabolism and does not affect the activity of the CYP system. Furthermore, AMI is highly selective for dopamine D2/D3 receptors; has minimal or no affinity for D1, D4, or D5 receptors. Despite the potential benefits of the combination of OLA and AMI, only a few open-label studies have been conducted, and no randomized clinical trial has been performed to date to examine the efficacy and tolerability of the combination. Hence, the goals of this study were to test the hypothesis that AMI augmentation would improve psychotic symptoms and be well tolerated in schizophrenic patients who showed poor response to OLA monotherapy.

**Objectives:**

The purpose of this study was to compare the efficacy and tolerability of continued olanzapine (OLA) versus amisulpride (AMI) augmentation in schizophrenic patients with poor response to OLA monotherapy.

**Methods:**

The present 4-week, randomized, rater-blinded study included 25 patients with schizophrenia who were partially or completely unresponsive to treatment with OLA monotherapy. Eligible subjects were randomly assigned at a 1:1 ratio to continuation of OLA monotherapy (OLA group) or OLA with AMI augmentation (AMI group). Efficacy was primarily evaluated using the Positive and Negative Syndrome Scale (PANSS) at baseline and at 1, 2, and 4 weeks.

**Results:**

The changes in PANSS total score and PANSS-positive subscale score were significantly different (p < 0.05) between the OLA and AMI groups. The differences between the two groups in PANSS-negative subscale, PANSS-general subscale, Brief Psychiatric Rating Scale, and Clinical Global Impression-Severity (CGI-S) scale scores were not statistically significant.

**Conclusions:**

AMI augmentation could be an effective strategy for patients with schizophrenia who show inadequate early response to OLA monotherapy.

**Disclosure of Interest:**

W.-M. Bahk Grant / Research support from: Handok Pharmaceuticals, Seoul, Korea, Y. S. Woo: None Declared, S.-Y. Park: None Declared, B.-H. Yoon: None Declared, S.-M. Wang: None Declared, M.-D. Kim: None Declared

